# Supplementary material for: Modeling glioblastoma heterogeneity as a dynamic network of cell states
Source: Mol Syst Biol. 2021 Sep 16;17(9):e10105. doi: 10.15252/msb.202010105 (PMC8444284; doi:10.15252/msb.202010105)
Supplement: Supplementary file 5 — Source Data for Figure 3 [file MSB-17-e10105-s001.zip › Figure3A_sourcedata/GSEA_3065/hallmarks_state4.GseaPreranked.1623416296068/HALLMARK_TNFA_SIGNALING_VIA_NFKB.html]

Details for gene set HALLMARK\_TNFA\_SIGNALING\_VIA\_NFKB[GSEA]

|  || Dataset | state4 |
| Phenotype | NoPhenotypeAvailable |
| Upregulated in class | na\_pos |
| GeneSet | HALLMARK\_TNFA\_SIGNALING\_VIA\_NFKB |
| Enrichment Score (ES) | 0.41603735 |
| Normalized Enrichment Score (NES) | 1.7002996 |
| Nominal p-value | 0.003717472 |
| FDR q-value | 0.019041955 |
| FWER p-Value | 0.064 |
Table: GSEA Results Summary

  

Fig 1: Enrichment plot: HALLMARK\_TNFA\_SIGNALING\_VIA\_NFKB      
 Profile of the Running ES Score & Positions of GeneSet Members on the Rank Ordered List

  

| PROBE | GENE SYMBOL | GENE\_TITLE | RANK IN GENE LIST | RANK METRIC SCORE | RUNNING ES | CORE ENRICHMENT || 1 | SAT1 |  |  | 4 | 0.669 | 0.0691 | Yes |
| 2 | CEBPD |  |  | 7 | 0.521 | 0.1231 | Yes |
| 3 | MARCKS |  |  | 27 | 0.363 | 0.1578 | Yes |
| 4 | PNRC1 |  |  | 30 | 0.354 | 0.1944 | Yes |
| 5 | IRS2 |  |  | 33 | 0.338 | 0.2293 | Yes |
| 6 | ID2 |  |  | 56 | 0.292 | 0.2563 | Yes |
| 7 | TUBB2A |  |  | 61 | 0.287 | 0.2855 | Yes |
| 8 | PTX3 |  |  | 238 | 0.188 | 0.2767 | Yes |
| 9 | NFE2L2 |  |  | 288 | 0.176 | 0.2871 | Yes |
| 10 | LITAF |  |  | 295 | 0.174 | 0.3043 | Yes |
| 11 | CDKN1A |  |  | 319 | 0.171 | 0.3184 | Yes |
| 12 | TSC22D1 |  |  | 369 | 0.160 | 0.3271 | Yes |
| 13 | SQSTM1 |  |  | 390 | 0.155 | 0.3401 | Yes |
| 14 | RCAN1 |  |  | 402 | 0.151 | 0.3541 | Yes |
| 15 | SNN |  |  | 407 | 0.151 | 0.3691 | Yes |
| 16 | BHLHE40 |  |  | 451 | 0.143 | 0.3771 | Yes |
| 17 | PHLDA1 |  |  | 464 | 0.142 | 0.3900 | Yes |
| 18 | JUNB |  |  | 490 | 0.139 | 0.4004 | Yes |
| 19 | NINJ1 |  |  | 582 | 0.127 | 0.3990 | Yes |
| 20 | BTG2 |  |  | 689 | 0.116 | 0.3939 | Yes |
| 21 | EIF1 |  |  | 695 | 0.115 | 0.4050 | Yes |
| 22 | SOCS3 |  |  | 701 | 0.114 | 0.4160 | Yes |
| 23 | FOS |  |  | 832 | 0.101 | 0.4055 | No |
| 24 | DENND5A |  |  | 853 | 0.099 | 0.4126 | No |
| 25 | TIPARP |  |  | 1345 | 0.069 | 0.3403 | No |
| 26 | HES1 |  |  | 1442 | 0.065 | 0.3315 | No |
| 27 | ZBTB10 |  |  | 1490 | 0.062 | 0.3304 | No |
| 28 | ABCA1 |  |  | 1654 | 0.057 | 0.3099 | No |
| 29 | RELA |  |  | 1693 | 0.055 | 0.3096 | No |
| 30 | TGIF1 |  |  | 1699 | 0.055 | 0.3145 | No |
| 31 | TNIP1 |  |  | 1925 | 0.049 | 0.2832 | No |
| 32 | B4GALT5 |  |  | 2065 | 0.045 | 0.2654 | No |
| 33 | CSF1 |  |  | 2180 | 0.042 | 0.2513 | No |
| 34 | TRIP10 |  |  | 2335 | 0.038 | 0.2304 | No |
| 35 | CEBPB |  |  | 2420 | 0.037 | 0.2206 | No |
| 36 | MCL1 |  |  | 2464 | 0.036 | 0.2173 | No |
| 37 | PDLIM5 |  |  | 2517 | 0.034 | 0.2125 | No |
| 38 | KDM6B |  |  | 2659 | 0.032 | 0.1930 | No |
| 39 | CD44 |  |  | 2687 | 0.031 | 0.1918 | No |
| 40 | PLPP3 |  |  | 2706 | 0.030 | 0.1921 | No |
| 41 | RIPK2 |  |  | 2812 | 0.028 | 0.1781 | No |
| 42 | TANK |  |  | 2890 | 0.027 | 0.1684 | No |
| 43 | RHOB |  |  | 2911 | 0.026 | 0.1679 | No |
| 44 | NAMPT |  |  | 3040 | 0.024 | 0.1497 | No |
| 45 | F3 |  |  | 3133 | 0.022 | 0.1372 | No |
| 46 | IFNGR2 |  |  | 3147 | 0.022 | 0.1374 | No |
| 47 | IL6ST |  |  | 3218 | 0.021 | 0.1282 | No |
| 48 | BCL6 |  |  | 3469 | 0.013 | 0.0891 | No |
| 49 | PPP1R15A |  |  | 3474 | 0.013 | 0.0898 | No |
| 50 | NFAT5 |  |  | 3567 | 0.010 | 0.0759 | No |
| 51 | VEGFA |  |  | 3787 | 0.001 | 0.0406 | No |
| 52 | KLF6 |  |  | 3920 | -0.005 | 0.0198 | No |
| 53 | BIRC2 |  |  | 3959 | -0.007 | 0.0144 | No |
| 54 | JUN |  |  | 3979 | -0.008 | 0.0121 | No |
| 55 | EGR1 |  |  | 3998 | -0.010 | 0.0102 | No |
| 56 | CFLAR |  |  | 4051 | -0.012 | 0.0031 | No |
| 57 | PDE4B |  |  | 4063 | -0.013 | 0.0027 | No |
| 58 | BTG1 |  |  | 4064 | -0.013 | 0.0040 | No |
| 59 | YRDC |  |  | 4081 | -0.014 | 0.0029 | No |
| 60 | SLC2A3 |  |  | 4169 | -0.020 | -0.0091 | No |
| 61 | ATF3 |  |  | 4262 | -0.026 | -0.0213 | No |
| 62 | BMP2 |  |  | 4398 | -0.036 | -0.0395 | No |
| 63 | PLK2 |  |  | 4403 | -0.036 | -0.0364 | No |
| 64 | JAG1 |  |  | 4496 | -0.044 | -0.0467 | No |
| 65 | MYC |  |  | 4618 | -0.053 | -0.0608 | No |
| 66 | G0S2 |  |  | 4644 | -0.055 | -0.0591 | No |
| 67 | FJX1 |  |  | 4678 | -0.058 | -0.0584 | No |
| 68 | FOSL1 |  |  | 4845 | -0.072 | -0.0778 | No |
| 69 | ETS2 |  |  | 4948 | -0.079 | -0.0861 | No |
| 70 | IRF1 |  |  | 5157 | -0.098 | -0.1096 | No |
| 71 | B4GALT1 |  |  | 5170 | -0.099 | -0.1012 | No |
| 72 | PLAUR |  |  | 5171 | -0.099 | -0.0909 | No |
| 73 | GADD45A |  |  | 5297 | -0.109 | -0.0998 | No |
| 74 | PTPRE |  |  | 5306 | -0.110 | -0.0897 | No |
| 75 | TNC |  |  | 5359 | -0.114 | -0.0861 | No |
| 76 | F2RL1 |  |  | 5406 | -0.119 | -0.0812 | No |
| 77 | ATP2B1 |  |  | 5594 | -0.144 | -0.0965 | No |
| 78 | CCNL1 |  |  | 5609 | -0.145 | -0.0836 | No |
| 79 | SDC4 |  |  | 5709 | -0.161 | -0.0828 | No |
| 80 | IER2 |  |  | 5719 | -0.164 | -0.0671 | No |
| 81 | SERPINE1 |  |  | 5812 | -0.186 | -0.0626 | No |
| 82 | LDLR |  |  | 5843 | -0.194 | -0.0473 | No |
| 83 | TNFAIP6 |  |  | 5872 | -0.199 | -0.0310 | No |
| 84 | BTG3 |  |  | 5895 | -0.206 | -0.0132 | No |
| 85 | PMEPA1 |  |  | 6009 | -0.241 | -0.0064 | No |
| 86 | CCND1 |  |  | 6203 | -0.454 | 0.0097 | No |
Table: GSEA details [plain text format]

  

Fig 2: HALLMARK\_TNFA\_SIGNALING\_VIA\_NFKB: Random ES distribution      
 Gene set null distribution of ES for **HALLMARK\_TNFA\_SIGNALING\_VIA\_NFKB**
